# Simulation-based education for cardiopulmonary resuscitation and airway management protocols: a brief report of a systematic review and meta-analysis

**DOI:** 10.1186/cc13672

**Published:** 2014-03-17

**Authors:** A Cortegiani, V Russotto, A Naro, G Sanzo, G Grutta, SM Raineri, A Giarratano

**Affiliations:** 1 University of Palermo, Italy

## Introduction

We aimed to summarize the efficacy of simulation-based education in cardiopulmonary resuscitation and airway management [1].

## Methods

We searched the MEDLINE, Scopus and EMBASE databases for all peer-reviewed articles enrolling physicians/medical students in a simulation of either cardiopulmonary resuscitation or airway management protocols compared with no intervention or traditional teaching methods. We categorized the outcomes of the studies into four groups: task success, process skill, time skill, knowledge. Task success was defined as evaluation of successful completion of the task, process skill as evaluation of the procedure, time skill as the time required to complete the task, and knowledge as the objective assessment of conceptual understanding. When studies investigated more than one outcome, we considered the primary outcome, the overall measure or the most clinically relevant outcome.

## Results

**Figure 1:**
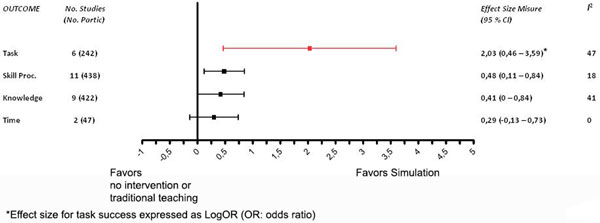


From 8,528 articles, we selected 24 studies (13 randomized controlled studies, eight pre-post studies, three case-control studies) involving 1,149 participants. Compared with no intervention or traditional teaching methods, simulation was associated with a significant improvement from mild to moderate of all outcomes (Figure 1). Log of odds ratio for task success was 2.03 (0.46 to 3.59) in favor of simulation. Pooled effect size for process skill was 0.48 (0.11 to 0.84), for time skill was 0.29 (0.13 to 0.73) and for knowledge was 0.41 (0 to 0.84).

## Conclusion

Simulation is an effective educational method to improve performance of physicians/medical students in the application of protocols for cardiopulmonary resuscitation and airway management.

## References

[B1] McGaghie WC A critical review of simulation-based medical education research: 2003-2009 Med Educ 2010 44 50 63 10.1111/j.1365-2923.2009.03547.x 20078756

